# Virtual Guided and Customized Orthognathic Surgery in Patients with Obstructive Sleep Apnea Syndrome: Accuracy and Clinical Outcomes

**DOI:** 10.3390/jcm14113780

**Published:** 2025-05-28

**Authors:** Marta Benito Anguita, Saad Khayat, Soledad López Martín, Natalia Bravo Quelle, Ignacio Navarro Cuéllar, Ana López López, José Luis Cebrián Carretero, José Luis del Castillo Pardo de Vera, Pablo Montes Fernández-Micheltorena, Manuel Tousidonis Rial, Giovanni Dell’Aversana Orabona, Farzin Falahat, José Zamorano León, Carlos Navarro Cuéllar

**Affiliations:** 1 Oral and Maxillofacial Surgery Department, University Hospital Gregorio Maranón, C/Dr. Esquerdo 46, 28007 Madrid, Spain; martabenito0910@hotmail.com (M.B.A.); saad.khayat@gmail.com (S.K.); ignacio.navarro@salud.madrid.org (I.N.C.); alopezl@salud.madrid.org (A.L.L.); manuel@tousidonisrial.com (M.T.R.); 2 Pneumology Department, University Hospital Gregorio Maranón, C/Dr. Esquerdo 46, 28007 Madrid, Spain; slopezmartin15@gmail.com; 3 Neurophysiology Department, University Hospital Gregorio Maranón, C/Dr. Esquerdo 46, 28007 Madrid, Spain; natalia.bravo@salud.madrid.org; 4 Oral and Maxillofacial Surgery Department, University Hospital La Paz, Paseo de la Castellana, 261, 28046 Madrid, Spain; josel.cebrian@salud.madrid.org (J.L.C.C.); joseldel.castillo@salud.madrid.org (J.L.d.C.P.d.V.); 5 Oral and Maxillofacial Surgery Department, University Hospital San Pedro, C/Piqueras 98, 26006 Logrono, Spain; pmontesf@riojasalud.es; 6 Maxillofacial Surgery Department, Università “Federico II”, 80131 Naples, Italy; giovanni.dellaversanaorabona@unina.it; 7 Maxillofacial Surgery Department, Hospital Clínico San Carlos, 28040 Madrid, Spain; ffalahat@yahoo.es; 8 Public Health and Maternal & Child Health Department, School of Medicine, Universidad Complutense, 28040 Madrid, Spain; jjzamorano@ucm.es; 9 Surgery Department, School of Medicine, Universidad Complutense de Madrid, 28040 Madrid, Spain

**Keywords:** orthognathic surgery, obstructive sleep apnea, CAD/CAM technology, virtual surgical planning, cutting guides, customized plates

## Abstract

**Background:** This preliminary case series aimed to evaluate the clinical and morphometric outcomes of maxillomandibular advancement (MMA) surgery in patients with severe obstructive sleep apnea (OSA) using virtual surgical planning (VSP), patient-specific cutting guides, and customized titanium plates. Primary outcomes included changes in the Apnea–Hypopnea Index (AHI), airway dimensions, surgical accuracy, and quality of life. **Methods**: In this preliminary case series, six patients with severe OSA underwent MMA surgery planned using three-dimensional VSP, and executed with the aid of CAD-/CAM-generated surgical guides and patient-specific osteosynthesis. Clinical variables included AHI, Epworth Sleepiness Scale (ESS), and computed tomography-based airway morphometry. Surgical accuracy was assessed by comparing planned and achieved skeletal movements. Statistical analysis was performed using Wilcoxon signed-rank tests and Spearman’s correlation. **Results**: The mean preoperative AHI decreased significantly from 48.8 ± 23.6 to 12.4 ± 10.0 (*p* = 0.035), and ESS scores improved from 14.5 ± 4.6 to 7.8 ± 2.1 (*p* = 0.029). Mean airway area increased significantly from 51.8 ± 9.0 mm^2^ to 91.8 ± 26.6 mm^2^ (*p* = 0.035). A strong but non-significant correlation was observed between airway gain and ESS improvement (*p* = 0.754, *p* = 0.084). No patients required CPAP at 6-month follow-up, and all were asymptomatic. The anteroposterior accuracy of skeletal movements was high: 82.6% for the maxilla and 85.8% for the pogonion, with mean absolute errors of 1.25 mm and 1.95 mm, respectively. Vertical accuracy was lower, particularly in the chin region, where error analysis showed greater variability. No statistically significant differences were found between planned and achieved movements in any vector. **Conclusions**: MMA surgery performed with VSP, cutting guides, and customized titanium plates offers a highly effective, safe, and precise treatment modality for selected OSA patients. This approach leads to a significant reduction in AHI, expansion of the upper airway, and improvement in patient-reported daytime functioning. High accuracy in skeletal repositioning—particularly in anteroposterior vectors—supports the reliability and reproducibility of digitally guided orthognathic surgery. These findings reinforce the role of technologically assisted MMA as a definitive treatment for severe OSA.

## 1. Introduction

Obstructive sleep apnea (OSA) is characterized by a total or partial collapse of the airway during sleep, leading to episodes of apnea–hypopnea. It is one of the most common sleep disorders, with a prevalence ranging from 4% to 38% [1,2]. The etiology of OSA is multifactorial. It is frequently associated with obesity, male gender, advanced age, smoking, alcohol consumption, and anatomical factors of the upper airway [3,4], such as mandibular retrognathia, tonsillar hypertrophy, nasal obstruction, or obesity [5].

OSA represents a significant burden on patient quality of life, workplace and automotive safety, and metabolic, cardiovascular, and neurocognitive health. The disease is characterized by repetitive cycles of upper airway collapse resulting from a lack of pharyngeal airway structural support and loss of muscle tone among upper airway dilators. Untreated severe OSA can have serious long-term consequences, including increased cardiovascular morbidity and mortality, insulin resistance, systemic hypertension, stroke, and neurocognitive decline. Chronic intermittent hypoxia and sleep fragmentation significantly impair quality of life, daytime functioning, and overall health outcomes, underscoring the need for definitive therapeutic strategies in non-compliant patients [3]. Polysomnography serves as the gold standard for diagnosis of OSA, and the Apnea–Hypopnea Index (AHI) is the most commonly used metric for quantifying disease severity [6].

Treatment is based on hygienic-dietary measures (weight loss, physical exercise) and the use of continuous positive airway pressure (CPAP) devices for patients with moderate to severe OSA. CPAP is well recognized as the most effective treatment for OSA, improving symptoms and reducing AHI. However, a disadvantage of CPAP is poor long-term patient adherence to treatment.

However, while CPAP remains the first-line treatment for OSA, alternative therapies play a crucial role in managing patients who cannot tolerate or do not respond to CPAP therapy. Another alternative for patients with OSA is the use of mandibular advancement devices (MADs), which are indicated for patients with mild to moderate OSA who do not tolerate CPAP. On the other hand, surgical treatment may be a viable option for patients with correctable anatomical factors in the upper airway [1,2,4,7].

Maxillomandibular advancement (MMA) surgery is performed through a Le Fort osteotomy in the maxilla and bilateral sagittal split osteotomy (BSSO) of the mandibular ramus. It increases the caliber of the upper airway and has been shown to be the most effective surgical option for the treatment of OSA, with a success rate of approximately 85% [2,4,8,9]. Orthognathic surgery, specifically MMA, is particularly indicated in patients with identifiable craniofacial anomalies (e.g., mandibular retrognathia, maxillary hypoplasia) contributing to airway obstruction, who are intolerant or non-adherent to CPAP therapy. These patients often derive the greatest benefit from skeletal advancement, as anatomical correction directly addresses the structural cause of airway collapse [9,10,11,12]. The exact amount of advancement and counterclockwise rotation of the maxillomandibular complex required to achieve significant airway widening and, consequently, a reduction in the AHI has not been precisely defined [10,11,12]. However, the magnitude of these movements is considerably greater compared to conventional orthognathic surgery. Thus, making it challenging to achieve the desired movements with accuracy and precision.

Virtual surgical planning (VSP) with cutting guides and customized plates provides enhanced precision and predictability in surgical outcomes. Few studies have investigated the role of computer-aided design/computer-aided manufacturing (CAD-CAM) technology and VSP in orthognathic surgery and patients with OSA [7,10]. VSP allows for a detailed and three-dimensional evaluation of the patient’s anatomical structures, facilitating the precise planning of the necessary movements to maximize the upper airway space. The use of CAD-CAM technologies, along with 3D printing, enables the creation of customized cutting guides and plates that are specifically tailored to the patient’s anatomy.

The aim of this study is to analyze the changes in the Apnea–Hypopnea Index (AHI), airway volume, and the accuracy of maxillary and mandibular movements, as well as their potential impact on patient-reported outcomes and daytime functioning in individuals with severe OSA treated with virtual surgical planning and customized orthognathic surgery.

## 2. Materials and Methods

To address the research purpose, this work was designed as a prospective observational case series conducted between 2022 and 2024. Six patients with OSA treated by MMA in the Oral and Maxillofacial Surgery Departments of Gregorio Marañón University Hospital (HGUGM) were considered for inclusion in this study. A specific informed consent form was designed. This study followed the Declaration of Helsinki on medical protocol, and the study and review of medical records, data collection, and subsequent analysis are endorsed by the Hospital Ethics Committee of Gregorio Marañón University Hospital (maxilohgugm 1/2022).

This study included patients who met all the following criteria:

- Patients with severe OSA diagnosed by polysomnography.
- Patients with poor tolerance to CPAP.
- Patients who underwent MMA with preoperative three-dimensional (3D) VSP and the use of cutting guides and customized osteosynthesis plates.
- Patients who underwent helical computed tomography (CT) six months after surgery.
- Patients who underwent polysomnography six months after surgery.

Patients who met any of the following criteria were excluded from this study:

- Patients with psychiatric disorders.
- Patients under 18 years of age.
- Syndromic patients.
- Patients who had undergone previous orthognathic surgery.
- Refusal to sign the informed consent.

All patients underwent a comprehensive preoperative evaluation, including medical history, physical examination, and anesthetic risk stratification.

### 2.1. Virtual Surgical Planning and Design of Cutting Guides and Customized Plates

**Figure 1:**
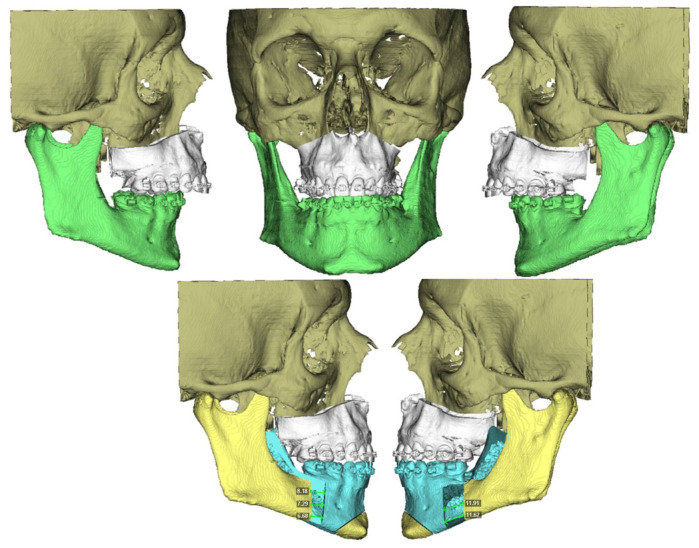
Step 1. The green mandible represents the preoperative mandibular position. The blue mandible ilustrates the postoperative (planned) position of the distal mandibular segment following virtual repositioning The yelow structures correspond to the cranial base and proximal mandibular segments, serving as stable reference points during simulation. The white maxilla and dentition are shown as unchanged anatomical references, used to guide occlusion and evaluate surgical accuracy.

Prior to surgery, helical CT scans were performed with the following parameters: 0.625 mm slice thickness, voxel size of 0.5 × 0.5 × 0.625 mm, 120 kVp, and 200 mA. Head and neck scans were acquired in natural head position with the patient in supine position. These scans, along with occlusal records obtained through digital models and clinical photographs of the patient, were integrated into the Dolphin Imaging surgical planning software version 11.95^®^. Using these records, a 3D reconstruction of the patient’s skull was created, aligned with its natural head position. The Le Fort and BSSO osteotomies were then designed. The planned movements of the maxilla and mandible were virtually simulated (Figure 1), and an intermediate and final splint were designed for a maxilla-first sequence treatment, which were then 3D printed.

After virtual surgical planning, CAD-CAM technology was used to design polyamide cutting guides in order to perform the osteotomies based on the planned movements, as well as customized titanium osteosynthesis plates. Both the cutting guides and the customized plates were produced using 3D printing (KLS MARTIN^®^, Tuttlingen, Germany).

**Figure 2:**
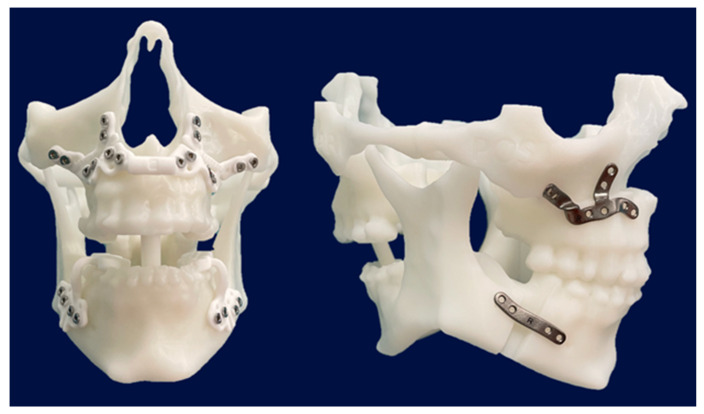
Step 2. Stereolithographic models showing final cutting guides and patient-specific titanium plates as part of the preoperative planning phase.

Additionally, stereolithographic (STL) models were printed at the 3D Printing Unit of the General University Hospital Gregorio Marañón to verify the position of the cutting guides, customized plates, and osteotomies during surgery (Figure 2).

The rationale for maxillomandibular advancement included skeletal class II with mandibular retrognathia and airway collapse confirmed on imaging and polysomnography. Genioplasty was planned in patients with additional aesthetic or skeletal indications (see below). The planning steps included CT segmentation, osteotomy simulation, airway evaluation, and the design of cutting guides and customized plates. Cutting guides and plates were fabricated by KLS Martin^®^ based on plans generated in Dolphin Imaging^®^. Airway volume was visualized during planning using Dolphin’s integrated simulation tools, but not quantitatively predicted preoperatively.

### 2.2. Surgical Technique

**Figure 3:**
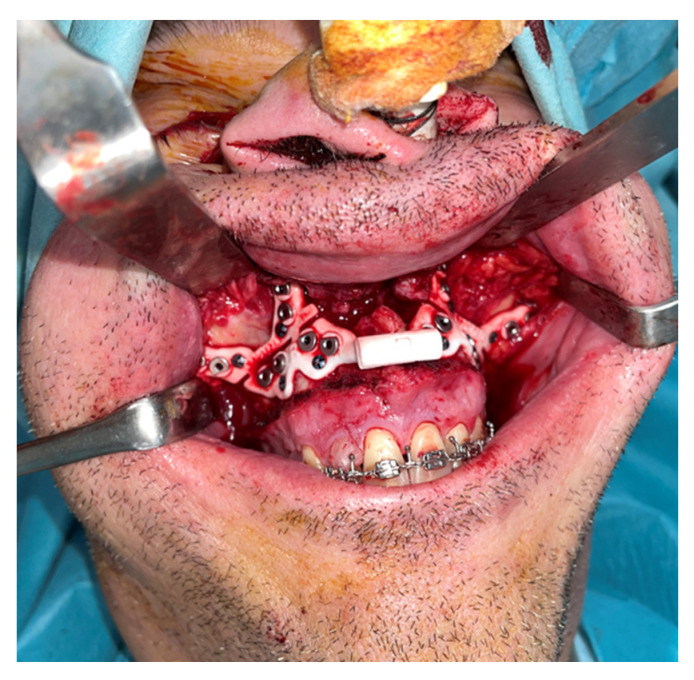
Step 3. Intraoral approach via vestibular sulcus incision and positioning of the maxillary bone-supported cutting guide, corresponding to the first surgical step following VSP.

An intraoral incision was designed in the upper vestibule, above the dental apices, extending from the right first molar to the left first molar. The mucoperiosteal flap was elevated to expose the maxilla. The bone-supported cutting guide was then positioned on the maxilla. Two cutting guides were designed, joined at the medial part, each with a tab for positioning on the piriform process. The cutting guide was designed to be fixed with 2.0 mm screws (Figure 3).

**Figure 4:**
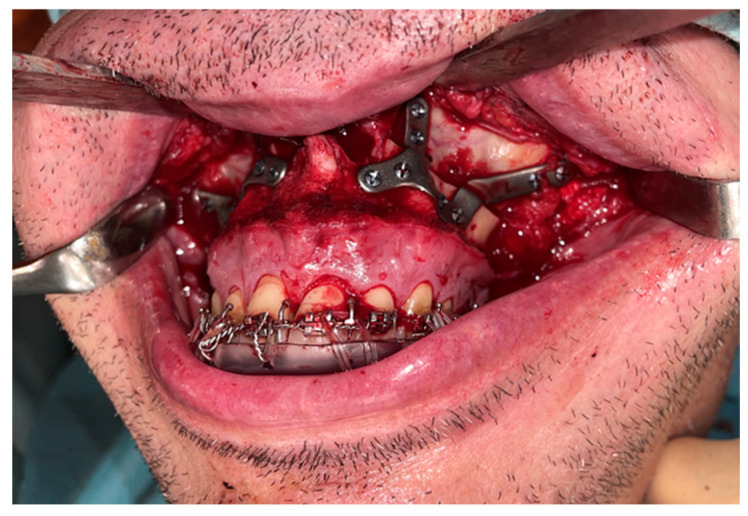
Le Fort I osteotomy and fixation of the maxilla using customized plates, verifying transfer of the virtual plan to the surgical site.

A Le Fort-type osteotomy was performed using a reciprocating saw. The maxilla was positioned according to the preoperative planning and fixed with customized titanium plates (Figure 4). The intermediate splint was used to perform a double check of the patient’s correct position of the maxilla and the dental occlusion.

**Figure 5:**
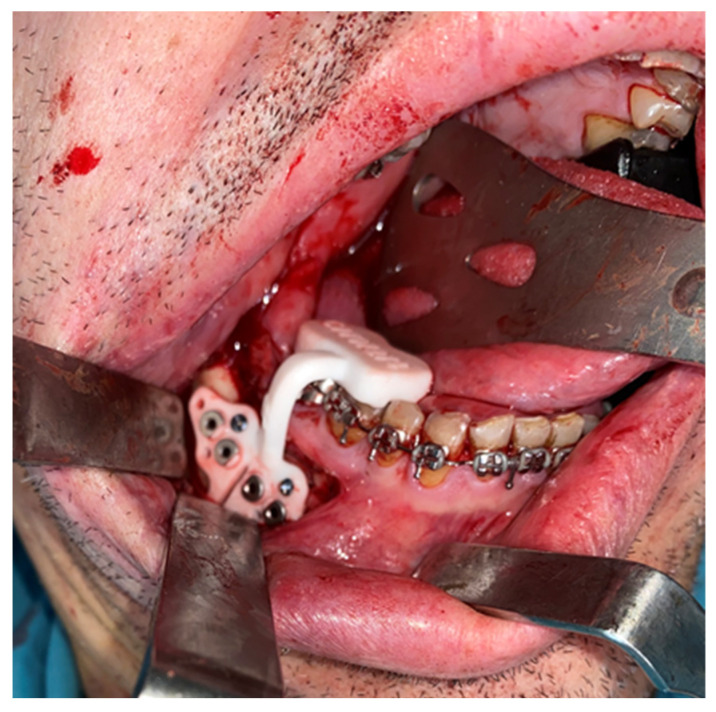
Placement of dento-osseous cutting guide for BSSO, aligned with the preoperative virtual plan.

Next, an incision was made in the vestibular mucosa of the mandibular ramus, along the oblique line, extending from the first molar to the anterior region of the mandibular ramus. A mucoperiosteal flap was elevated to expose the external surface of the mandibular ramus, identifying the inferior alveolar nerve. The dento-osseous-supported cutting guide was positioned, and the BSSO osteotomy was performed using a reciprocating saw (Figure 5).

**Figure 6:**
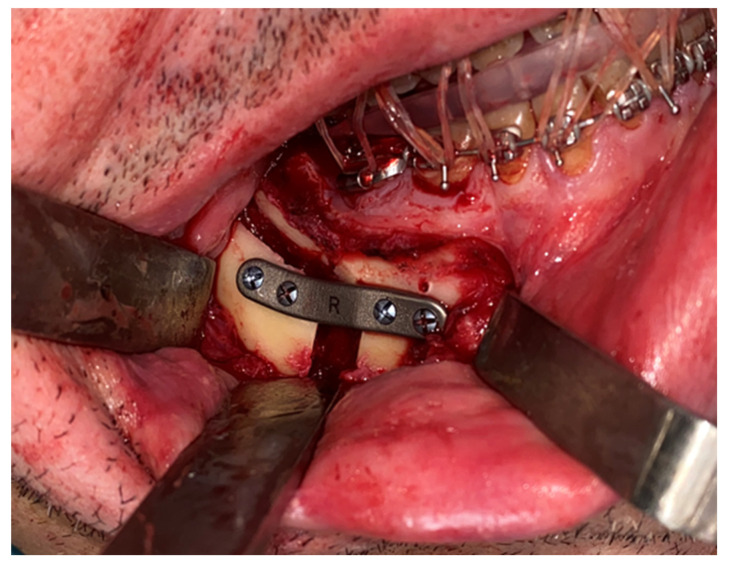
Mandibular repositioning and fixation using custom-designed titanium plates, confirming intraoperative execution of the virtual plan.

The same procedure was performed on the contralateral side. The mandible was positioned according to the preoperative plan and fixed with customized titanium plates (Figure 6). The final splint was used to perform a double check of the patient’s correct occlusion.

All patients received antibiotic prophylaxis with amoxicillin-clavulanic acid, which was continued orally for up to seven days postoperatively.

### 2.3. Study Variables

The variables recorded include gender, age, smoking habit, obesity, skeletal class, surgery duration, genioplasty, postoperative complications, length of hospital stay, AHI (pre- and postoperative), Epworth Sleepiness Scale score (pre- and postoperative), upper airway volume (pre- and postoperative), use of CPAP at 6 months post-surgery, movement of the upper incisor in the anteroposterior (AP) plane, planned movement of the upper incisor in the AP plane, movement of the upper incisor in the vertical plane, planned movement of the upper incisor in the vertical plane, post-surgical distance between the upper incisor and the vertical plane at the glabella, planned distance between the upper incisor and the vertical plane at the glabella, post-surgical distance between the pogonion and the vertical plane at the glabella, and planned distance between the pogonion and the vertical plane at the glabella.

### 2.4. Airway Volume Assessment

Upper airway volumes were assessed using 3D CT scans performed pre- and 6 months postoperatively. Segmentation of the airway was performed using Dolphin Imaging software^®^. Threshold-based volumetric analysis was applied using Hounsfield Unit (HU) ranges of −1024 to −300. Anatomical landmarks for the segmentation were the posterior nasal spine (PNS) superiorly and the base of the epiglottis inferiorly. The minimum cross-sectional area and total volume were recorded.

### 2.5. Statistical Analysis

Categorical variables were expressed as frequency and percentage, while quantitative variables were expressed as mean and mean standard deviation (Mean ± MSD). Normality was assessed using the Shapiro–Wilk test and visual inspection of Q-Q plots. Due to the small sample size and observed deviations from normality in several variables, non-parametric tests (Wilcoxon signed-rank and Spearman correlation) were applied to minimize the risk of type I error due to assumption violations. The Wilcoxon signed-rank test was used to compare pre- and post-surgical values of the Apnea–Hypopnea Index (AHI), Epworth Sleepiness Scale scores, and upper airway space. Surgical accuracy was evaluated by comparing the planned skeletal displacements of anatomical landmarks with the actual postoperative outcomes. Four measurements were analyzed: anteroposterior (AP) and vertical movements of both the upper central incisor (representing the maxilla) and the pogonion (representing the mandible). Planned movements were extracted from virtual surgical planning files, while obtained movements were measured from standardized postoperative lateral cephalometric radiographs using stable cranial base structures for superimposition.

Accuracy was assessed by calculating the mean absolute error (MAE), defined as the absolute difference in millimeters between planned and obtained movements for each case. The mean planned movement and mean obtained movement (expressed as absolute magnitudes) were also reported to characterize the overall extent of displacement. Additionally, a percentage accuracy metric was calculated using the formula:

$$Accuracy\;\left(\%\right)=100-(\frac{MAE}{Mean\;Planned\;Movement}\times 100)$$

This percentage was interpreted with caution, particularly in cases where the planned movement was 0 mm, as relative error becomes undefined or exaggerated in such instances. In these cases—most notably in the vertical pogonion measurements—accuracy percentage was deemed mathematically unstable and excluded from interpretation. To provide a clinically meaningful perspective, an alternative threshold-based analysis was performed, using the proportion of cases in which the obtained movement deviated by no more than ±1.0 mm from the plan was calculated and reported as the clinical accuracy. Spearman’s rank correlation coefficient was used to evaluate the relationship between pre- and postoperative values.

The level of statistical significance was set at *p* < 0.05 for all comparisons. All statistical analyses were performed using IBM SPSS Statistics for Windows, Version 26.0 (IBM Corp., Armonk, NY, USA).

## 3. Results

A total of six patients met the inclusion and exclusion criteria. The mean age was 48.5 years, and all patients included in this study were male. Only one patient was a smoker (16.67%), and none were obese. None of the patients presented systemic diseases such as diabetes mellitus, cardiovascular pathology, or active psychiatric illness. All were classified as ASA I or II. All patients were classified as skeletal class II. The mean surgery duration was 220 min. Genioplasty was included in four cases (66.67%) based on preoperative facial aesthetics (e.g., deficient chin projection). In one of those cases, it was to address potential airway gain by anterior repositioning of the genial tubercle. This was planned according to patient-specific aesthetic goals and not airway simulation. No complications were observed in the immediate or late postoperative period. The mean length of hospital stay was 1.5 days (Table 1). All patients underwent paired CT scans pre- and postoperatively for direct volumetric comparison using 3D reconstructions.

**Figure 7:**
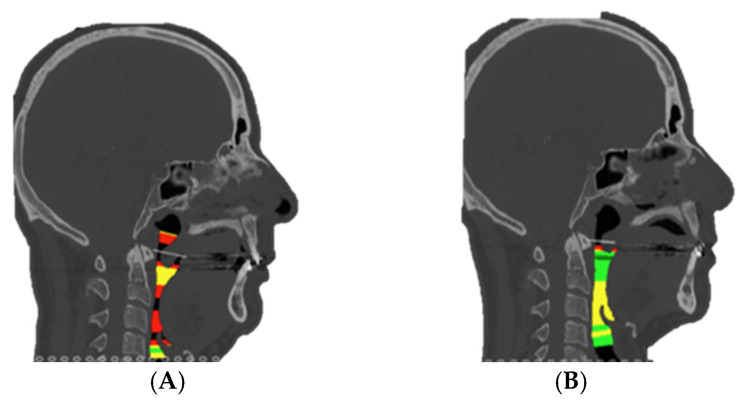
Comparison of preoperative upper airway volume (**A**) (minimum area: 46 mm^2^) versus postoperative upper airway volume (**B**) (minimum volume: 67 mm^2^), showing representative volumetric improvement.

Postoperative clinical outcomes showed consistent improvements across key respiratory and subjective measures (Table 2). Descriptive and inferential analyses demonstrated significant postoperative improvements across all measured parameters. The Apnea–Hypopnea Index (AHI) decreased from a preoperative mean of 48.8 ± 23.6 to 12.4 ± 10.0 postoperatively (Z = −2.201, *p* = 0.035; 95% CI [0.031–0.038]). Similarly, the Epworth Sleepiness Scale improved from 14.5 ± 4.6 to 7.8 ± 2.1 (Z = −2.207, *p* = 0.029; 95% CI [0.026–0.033]), indicating a substantial decrease in subjective daytime sleepiness. Airway space increased from a preoperative mean of 51.8 ± 9.0 mm^2^ to 91.8 ± 26.6 mm^2^ postoperatively (Z = −2.201, *p* = 0.035; 95% CI [0.031–0.038]) (Figure 7). Spearman’s rank correlation analysis revealed a strong positive association between airway space enlargement and improvement in Epworth scores (ρ = 0.754, *p* = 0.084). This result may suggest that anatomical gains may be linked to subjective relief from daytime sleepiness. No significant correlations were found between AHI improvement and either airway change (ρ = −0.314, *p* = 0.544) or Epworth reduction (ρ = −0.348, *p* = 0.499), possibly due to the limited sample size (Table 2). None of the patients were using CPAP at 6 months post-surgery and all of them were asymptomatic.

In terms of skeletal movement accuracy, both maxillary and mandibular results demonstrated high fidelity to the virtual plan, especially in the anteroposterior plane (Table 3). The comparison between planned and achieved surgical movements revealed variable concordance across anatomical regions. In the anteroposterior (AP) plane, the maxilla showed a mean planned movement of 7.17 mm and a mean obtained movement of 6.82 mm. The mean absolute error was 1.25 mm and the accuracy was 82.6%. A total of 50% of cases were within a clinically acceptable threshold of ±1.0 mm.

The pogonion exhibited a mean planned advancement of 13.73 mm and a mean obtained movement of 12.62 mm, with a mean absolute error of 1.95 mm and an accuracy of 85.8%, also achieving 50% clinical agreement.

In the vertical plane, the maxilla demonstrated a mean planned movement of 2.00 mm and a mean obtained movement of 2.22 mm, with a mean absolute error of 1.02 mm and an accuracy of 49.0%; again, 50% of cases were within ±1.0 mm of the intended target.

Conversely, the vertical movement of the pogonion presented the poorest agreement, with a mean planned movement of 2.05 mm and a mean obtained displacement of 4.07 mm, resulting in a mean absolute error of 3.02 mm and a calculated accuracy of −26.9%. However, due to the inclusion of planned values equal to zero, this accuracy metric becomes mathematically unstable and clinically misleading. In such cases, relative error is undefined or artificially inflated, and thus, percentage-based accuracy should not be interpreted. Instead, deviations from the surgical plan should be assessed using absolute error values and threshold-based clinical criteria to more accurately reflect surgical performance and outcome fidelity. Additionally, there were no differences between planned and obtained movements in any of the measured directions: upper incisor anteroposterior (Monte Carlo *p* = 0.218; 95% CI [0.209–0.226]), upper incisor vertical (*p* = 0.719; 95% CI [0.710–0.728]), pogonion anteroposterior (*p* = 0.441; 95% CI [0.431–0.451]), and pogonion vertical (*p* = 0.688; 95% CI [0.678–0.697]). (Table 3).

## 4. Discussion

OSA is one of the most common sleep disorders, with a prevalence ranging from 4% to 38% across different studies [1,2]. Moreover, OSA is considered a significant cardiovascular risk factor. During sleep, the airway caliber decreases, and its resistance to airflow increases. Consequently, blood pressure must rise to compensate for repeated episodes of hypoxia and the increased respiratory effort during sleep, which can lead to complications such as coronary artery disease, hypertension, cardiac arrhythmias, or cerebrovascular accidents. Additionally, OSA increases the risk of diabetes, as this stress condition results in elevated cortisol release [7]. For this reason, early diagnosis and treatment are of vital importance to prevent potentially severe cardiovascular events.

Diagnosis is established through polysomnography. An AHI greater than 15 in asymptomatic individuals, or greater than 5 in those exhibiting symptoms such as daytime sleepiness, non-restorative sleep, excessive fatigue, or sleep-related quality-of-life impairment, confirms the presence of OSA [1,7].

Treatment is based on hygienic-dietary measures and the use of CPAP for patients with moderate to severe OSA. However, this device is uncomfortable for many patients, leading to generally poor adherence to treatment.

Another non-surgical treatment option is MAD, indicated for patients with mild to moderate OSA and those who do not tolerate or adequately respond to CPAP therapy. It may also be considered in patients with severe OSA who are unable to use CPAP, although its effectiveness is generally lower compared to CPAP [11,12,13,14,15,16]. The procedure involves the use of an oral device that advances the mandible forward, increasing the upper airway caliber and reducing its collapsibility during sleep. MADs can be either prefabricated or custom-made, with the latter being more effective and better tolerated [15]. These devices contribute to a significant reduction in the AHI and improvement in oxygen saturation, and are better tolerated than CPAP, which may translate into better long-term clinical outcomes. However, they are less effective than CPAP in reducing the AHI, particularly in severe cases [13,15,16].

Surgical treatment of OSA includes various options, each with its own benefits and drawbacks compared to MMA surgery. The American Academy of Sleep Medicine recommends considering surgical treatment in patients who are intolerant to CPAP and have a body mass index (BMI) of less than 40 kg/m^2^ [4,17].

First, uvulopalatopharyngoplasty is a surgical procedure involving the removal of the uvula, part of the soft palate, and redundant mucosa from the palatal arches, as well as the tonsils, with the goal of widening the upper airway. This procedure is less invasive than MMA and has been shown to reduce the AHI in approximately 50% of patients. However, uvulopalatopharyngoplasty does not always normalize AHI and may be insufficient in cases of moderate to severe OSA [17]. Additionally, it can be associated with postoperative complications such as pain, difficulty swallowing, and voice changes [18].

Genioglossus advancement, on the other hand, repositions the genioglossus muscle anteriorly to increase tension at the tongue base. This technique is specifically indicated for cases involving retrolingual airway collapse and can be combined with other procedures. However, it is less effective than MMA in severe cases and may require additional interventions [19,20]. Although genioplasty may contribute to improved airway patency by advancing tongue muscle attachments, in our series, it was primarily indicated for aesthetic and profile balance.

Hyoid suspension is a surgical procedure that repositions the hyoid bone to increase tension at the tongue base, thereby expanding the upper airway, particularly the retrolingual space. It may be beneficial in combination with other procedures, but is less effective as a standalone treatment and may not address all sites of airway collapse [19,20].

Multilevel surgery involves the combination of multiple surgical procedures to target various sites of upper airway obstruction. These may include uvulopalatopharyngoplasty, genioglossus advancement, hyoid suspension, tongue base reduction, and other interventions directed at the nasal and hypopharyngeal regions. Multilevel surgery can be more effective than single procedures in complex cases; however, it entails greater surgical complexity and a higher risk of complications. Although multilevel surgery may be less invasive and associated with a lower rate of major complications (1.1% vs. 3.2% for MMA), its effectiveness is inferior to MMA in terms of AHI reduction, success rates, and cure rates [2].

MMA orthognathic surgery has been widely documented as an effective therapeutic option for patients with moderate to severe OSA who have poor tolerance to CPAP, or in patients who request a reliable alternative to the use of CPAP. The primary mechanism of this surgery is based on advancing the maxillomandibular complex, which increases the volume of the upper airway (retropalatal and retrolingual space) [10] and significantly reduces the AHI. Previous studies have demonstrated that this procedure has a success rate exceeding 85%, with objective improvements in sleep architecture and patient quality of life [2,4,8,21].

In our study, the findings confirm the effectiveness of MMA in reducing the AHI and increasing upper airway volume. Although the accuracy of skeletal movements was assessed, the primary clinical objective of MMA in OSA remains functional improvement—namely, reduction in AHI and increase in airway volume. These metrics were therefore emphasized as primary endpoints in our outcome analysis. Specifically, the decrease in AHI from 48.8 to 12.4 episodes per hour and the increase in airway volume from 51.8 to 91.8 mm^2^ are consistent with the previous literature supporting MMA as a first-line intervention in selected patients. The surgical success rate is defined as a reduction in AHI by 50% and an AHI of fewer than 20 episodes per hour after surgery [9]. In our study, the six patients who underwent surgery using VSP, cutting guides and customized plates, a reduction in AHI of more than 50% was achieved, which represents a 100% success rate. On the other hand, in five of the six patients, a reduction in AHI of fewer than 20 apneas was achieved (83.33%).

Moreover, the reduction in the Epworth Sleepiness Scale score from 14.5 to 9 indicates a subjective improvement in daytime sleepiness, a key marker of surgical success. A strong positive correlation was observed between airway space enlargement and the improvement in Epworth scores. This suggests that anatomical airway gains may directly contribute to enhanced patient-reported outcomes

Achieving accuracy and predictability in outcomes presents a challenge in orthognathic surgery [22]. In conventional orthognathic surgery, the mandible and maxilla are operated on sequentially using a semi-adjustable articulator and model surgery to obtain intermediate and final splints. However, this method can introduce errors at multiple stages and does not accurately reflect the natural head position, making it difficult to predict postoperative outcomes [7]. Currently, VSP enhances precision and treatment customization, allowing for preoperative simulation of results [23,24]. However, the use of occlusal splints to transfer this planning to the surgical site may not be precise enough to reproduce the planned movements exactly, especially in the vertical plane. Additionally, achieving stable and precise intermaxillary fixation to guide movements can be challenging in patients with severe discrepancies or extensive planned movements.

On the other hand, the use of conventional osteosynthesis systems may not provide sufficient stability to counteract the influence of muscular forces on the osteotomized fragments. Therefore, the integration of VSP with CAD/CAM technology for designing cutting guides and high-rigidity custom-made miniplates has improved the precision of MMA [22,25]. However, our results indicate that while an accuracy of 85.8% was achieved in the anteroposterior movement of the pogonion, accuracy in the vertical plane could not be reliably calculated due to the presence of planned displacements equal to zero in some cases. This discrepancy may be attributed to the inherent biomechanical limitations of the bilateral sagittal split osteotomy (BSSO) and the influence of soft tissues on postoperative stability. In such instances, relative error becomes mathematically unstable or artificially inflated, rendering percentage-based accuracy misleading. Thus, interpretation should shift from accuracy percentages toward evaluating whether movements fall within clinically acceptable error margins, typically ±1.0 mm. In this context, 50% of patients achieved vertical pogonion positioning within this threshold. Similarly, the upper incisor showed an accuracy of 82.6% in the anteroposterior plane and 49.0% in the vertical plane, with half of the cases again within ±1.0 mm. Despite these deviations, there were no statistically significant differences between planned and obtained movements in any direction, reinforcing the overall fidelity of the virtual planning. Importantly, it appears that deviations in vertical positioning—particularly within narrow clinical margins—may not significantly affect functional outcomes. Therefore, while vertical control is relevant for occlusion and aesthetics, its role in symptom relief and airway improvement may be less critical than precise anteroposterior advancement. Regardless, further optimization of surgical planning and execution protocols is needed.

In terms of safety, MMA is generally well tolerated, although it may require hospitalization and postoperative monitoring in intermediate care units, depending on the patient’s individual risk [26]. Major complications are rare but can occur, highlighting the necessity of performing the procedure in a hospital setting with intensive care availability.

To date, this is the first study to evaluate changes in the Apnea–Hypopnea Index (AHI), airway volume, surgical accuracy of maxillary and mandibular movements, and patient-reported outcomes in patients with severe OSA treated with virtual surgical planning and customized orthognathic surgery.

These preliminary results indicate that the combination of MMA with virtual planning and customized devices improves surgical predictability and may contribute to more consistent clinical outcomes. Additional studies with larger sample sizes and comparisons with traditional techniques are needed to further validate these findings and optimize surgical protocols.

The incorporation of VSP, patient-specific cutting guides, and customized titanium plates into MMA surgery represents a major technological advancement in the surgical management of OSA. This personalized approach offers significant advantages over conventional techniques, particularly in terms of clinical outcomes, surgical accuracy, and anatomical predictability [27].

One of the most clinically relevant benefits is the significant reduction in the AHI observed in patients treated with this technology-driven methodology. By enabling precise control over the magnitude and direction of skeletal movements, VSP allows for a more strategic advancement of the maxillomandibular complex, resulting in optimal tensioning of the pharyngeal soft tissues. This leads to a more effective and consistent expansion of the upper airway, contributing to the reduction in airway collapsibility during sleep. Numerous studies have validated the relationship between greater skeletal advancement and postoperative AHI reduction, and our findings further confirm that digitally assisted MMA surgery enhances this therapeutic effect.

Helical CT was selected over CBCT due to its superior soft tissue contrast, which is essential for accurate segmentation and volumetric analysis of the upper airway. Given that airway volume was a primary outcome of this study, the higher resolution and broader anatomical coverage offered by helical CT scans justified their use despite the higher radiation dose compared to CBCT. Morphometric analysis based on pre- and postoperative imaging has demonstrated marked increases in upper airway volume and minimum cross-sectional area, particularly in the retropalatal and retroglossal spaces. These volumetric gains are closely associated with improved respiratory parameters and reduced airway resistance. The enhanced airway patency achieved through personalized MMA contributes not only to better sleep-related breathing but also to a perceived improvement in patients’ daytime functioning and sleep satisfaction, as reflected by reductions in Epworth scores, including reductions in daytime somnolence, improved neurocognitive function, and heightened overall well-being.

In addition to functional benefits, aesthetic outcomes are often improved due to the ability to simulate and fine-tune the final facial profile during the virtual planning process. This dual focus on airway optimization and facial harmony is particularly relevant in patients with retrognathic or hypoplastic facial patterns, where MMA serves both therapeutic and cosmetic purposes.

Another important consideration is the long-term skeletal stability of the mandibular advancement. Customized titanium plates designed specifically for each patient’s anatomy allow for rigid fixation that conforms perfectly to the bony surfaces, minimizing micromovements and promoting optimal healing. Early evidence suggests that this degree of individualization may contribute to superior long-term positional stability compared to conventional plates or intraoperative hand-bent miniplates. Follow-up beyond the six-month period will be critical to assess the long-term stability of airway expansion and the durability of functional improvements, and it is planned as part of our ongoing protocol.

When comparing our outcomes with those reported in studies using traditional MMA techniques, the precision and reproducibility offered by VSP and patient-specific devices appear to translate into improved airway metrics and more predictable results. Conventional methods, while effective, often rely heavily on intraoperative judgment and 2D planning, which introduces variability and may limit the extent of advancement achievable with safety and symmetry.

Despite these advantages, it is important to acknowledge the limitations of this technology-driven approach. First, the cost associated with VSP, custom guides, and plates can be significantly higher than standard procedures, potentially limiting access in certain healthcare systems or regions. Second, the availability of advanced imaging, planning software, and manufacturing infrastructure is not universal, particularly in resource-limited settings. Lastly, there exists a notable learning curve associated with mastering the digital workflow, which may initially increase preoperative planning time and require training for both surgeons and technical teams.

## 5. Study Limitations

This work should be interpreted as a preliminary case series. As such, it provides early insights but is limited in its generalizability due to the small sample size and absence of a control group treated with conventional techniques, which prevents direct comparisons regarding the differential efficacy of VSP compared to traditional surgical planning. Another limitation is the homogeneity of the sample, which included only male patients. Although this reflects the higher prevalence of severe OSA in men, the lack of gender diversity restricts extrapolation to the broader patient population. Inclusion of female patients in future studies will be essential to determine whether these outcomes apply across sexes.

## 6. Conclusions

In summary, this preliminary case series demonstrates that maxillo-mandibular advancement with VSP, cutting guides, and customized titanium plates provides a highly effective, safe, and precise intervention for selected OSA patients, offering both functional and structural improvements that support its growing role as a definitive treatment option. This technologically assisted approach enables highly precise maxillary and mandibular movements, ensuring accurate skeletal positioning, particularly in the anteroposterior movements. The integration of these techniques within our workflow leads to a substantial reduction in the Apnea–Hypopnea Index (AHI), reflecting improved respiratory function during sleep. Furthermore, three-dimensional analysis confirms a notable increase in upper airway volume. Beyond objective respiratory metrics, patients benefit from a marked enhancement in quality of life, with reported improvements in daytime somnolence, cognitive performance, and overall sleep satisfaction. Future studies with longer follow-up periods are necessary to assess the durability of airway and quality-of-life improvements observed at six months.

## Figures and Tables

**Table 1:** Variables and categories.

Variable	Category	Total (% or Mean)
Age (years)		48.5 ± 8.7
Gender	Male	6 (100%)
	Female	0 (0%)
Smoking habit	Yes	1 (16.67%)
	No	5 (83.33%)
Obesity	Yes	0 (0%)
	No	6 (100%)
Skeletal class	I	0 (0%)
	II	6 (100%)
	III	0 (0%)
Surgery duration (minutes)		251.8 ± 37.7
Genioplasty	Yes	4 (66.67%)
	No	2 (33.33%)
Postoperative complications	Yes	0 (0%)
	No	6 (100%)
Length of the hospital stay (days)		1.5 ± 0.3

**Table 2:** (**A**) Preoperative and postoperative values of AHI, Epworth Sleepiness Scale scores, and superior airway space, along with their corresponding differences (Δ). (**B**) Spearman correlation analysis revealed a strong positive association between improvements in airway space and reductions in Epworth scores (ρ = 0.754, *p* = 0.084), while changes in AHI did not significantly correlate with other variables. Values are presented as means ± standard deviation unless otherwise indicated. (**C**) Wilcoxon signed-rank tests demonstrated statistically significant postoperative improvements in all three variables: AHI (*p* = 0.035; 95% CI [0.031–0.038]), Epworth scores (*p* = 0.029; 95% CI [0.026–0.033]), and airway space (*p* = 0.035; 95% CI [0.031–0.038]).

(A)									
Patient	Preoperative AHI	Postoperative AHI	*δ*	Preoperative Epworth	Postoperative Epworth	*δ*	Preoperative Superior Airway SPACE (Minimum Axial Area) (mm^2^)	Postoperative Superior Airway SPACE (Minimum Axial Area) (mm^2^)	*δ*
1	71	32	39	15	9	6	56	84	28
2	25	12	13	13	7	6	42	73	31
3	35	11	24	17	10	7	53	141	88
4	67	4.8	62.2	18	9	9	47	86	39
5	72	7.7	64.3	6	4	2	46	67	21
6	23	7	16	18	8	10	67	100	33
**MEAN ± SD**	**48.8 ± 23.6**	**12.4 ± 10.0**		**14.5 ± 4.6**	**7.8 ± 2.1**		**51.8 ± 9.0**	**91.8 ± 26.6**	
(**B**)									
		***δ*** **AHI**			***δ*** **Epworth**			***δ*** **Airway Space**	
***δ*** **AHI**		1			−0.348			−0.314	
*p* VALUE					0.499			0.544	
***δ*** **EPWORTH**		−0.348			1			**0.754**	
*p* VALUE		0.499						0.084	
***δ*** **AIRWAY SPACE**		−0.314			**0.754**			1	
*p* VALUE		0.544			0.084				
(**C**)									
		**AHI**			**Epworth**			**Airway Space**	
Z VALUE		−2.201			−2.207			−2.201	
*p* VALUE (Exact, 2-tailed)		0.035			0.029			0.035	
95% CI (2-tailed)		[0.031–0.038]			[0.026–0.033]			[0.031–0.038]	

**Table 3:** Accuracy and statistical comparison of planned versus obtained skeletal movements in the anteroposterior and vertical planes for the upper incisor (maxilla) and pogonion (mandible). Wilcoxon signed-rank tests were used to compare planned and obtained values for each vector. No statistically significant differences were found in any direction (*p* > 0.05). **Note**: Accuracy could not be reliably calculated for the pogonion in the vertical plane due to the inclusion of planned values equal to zero. In such cases, relative error is mathematically undefined or inflated, and percentage-based accuracy becomes unstable and clinically misleading. Evaluation should instead rely on absolute error values and threshold-based clinical criteria.

Patient	Movement of the Upper Incisor in the AP Plane (mm)	Planned Movement of the Upper Incisor in the AP Plane (mm)	Movement of the Upper Incisor in the Vertical Plane (mm)	Planned Movement of the Upper Incisor in the Vertical Plane (mm)	Movement of the Pogonion in the AP Plane (mm)	Planned Movement of the Pogonion in the AP Plane (mm)	Movement of the Pogonion in the Vertical Plane (mm)	Planned Movement of the Pogonion in the Vertical Plane (mm)
1	+8.0	+10.0	+1.6	+2.0	+10.0	+10.8	−7.0	−5.7
2	+5.8	+5.0	+2.8	+4.0	+16.5	+14.0	+1.0	+4.4
3	+6.4	+8.0	+1.2	+2.0	+8.5	+10.0	+5.0	0
4	+9.4	+10	+3.7	+2.0	+13.9	+14.8	0	+0.6
5	+7.5	+7.0	+2.4	+2.0	+12.8	+12.8	+2.7	0
6	+1.0	+3.0	−1.6	+0	+14.0	+20.0	−8.7	−3.6
**Movement**			**Z Value**		***p*** **Value**		**95% CI**	
**UPPER AP**			−1.367		0.218		[0.209–0.226]	
**UPPER VERTICAL**			−0.420		0.719		[0.710–0.728]	
**POGONION AP**			−0.944		0.441		[0.431–0.451]	
**POGONION VERTICAL**			−0.524		0.688		[0.678–0.697]	
**MOVEMENT**			**Mean Planned (mm)**		**Mean Absolute Error**		**Accuracy %**	
**UPPER AP**			7.17		1.25		82.6	
**UPPER VERTICAL**			2.00		1.02		49.0	
**POGONION AP**			13.73		1.95		85.8	
**POGONION VERTICAL**			2.05		3.02		- (SEE NOTE)	

## Data Availability

The data presented in this study are available on request from the corresponding author. The data are not publicly available due to data protection regulations.
